# From learning taxonomies to phylogenetic learning: Integration of 16S rRNA gene data into FAME-based bacterial classification

**DOI:** 10.1186/1471-2105-11-69

**Published:** 2010-01-30

**Authors:** Bram Slabbinck, Willem Waegeman, Peter Dawyndt, Paul De Vos, Bernard De Baets

**Affiliations:** 1 Laboratory of Microbiology, Ghent University, K.L. Ledeganckstraat 35, 9000 Ghent, Belgium; 2 KERMIT, Department of Applied Mathematics, Biometrics and Process Control, Ghent University, Coupure links 653, 9000 Ghent, Belgium; 3 Department of Applied Mathematics and Computer Science, Ghent University, Krijgslaan 281 S9, 9000 Ghent, Belgium; 4 BCCM™/LMG Bacteria Collection, K.L. Ledeganckstraat 35, 9000 Ghent, Belgium

## Abstract

**Background:**

Machine learning techniques have shown to improve bacterial species classification based on fatty acid methyl ester (FAME) data. Nonetheless, FAME analysis has a limited resolution for discrimination of bacteria at the species level. In this paper, we approach the species classification problem from a taxonomic point of view. Such a taxonomy or tree is typically obtained by applying clustering algorithms on FAME data or on 16S rRNA gene data. The knowledge gained from the tree can then be used to evaluate FAME-based classifiers, resulting in a novel framework for bacterial species classification.

**Results:**

In view of learning in a taxonomic framework, we consider two types of trees. First, a FAME tree is constructed with a supervised divisive clustering algorithm. Subsequently, based on 16S rRNA gene sequence analysis, phylogenetic trees are inferred by the NJ and UPGMA methods. In this second approach, the species classification problem is based on the combination of two different types of data. Herein, 16S rRNA gene sequence data is used for phylogenetic tree inference and the corresponding binary tree splits are learned based on FAME data. We call this learning approach 'phylogenetic learning'. Supervised Random Forest models are developed to train the classification tasks in a stratified cross-validation setting. In this way, better classification results are obtained for species that are typically hard to distinguish by a single or flat multi-class classification model.

**Conclusions:**

FAME-based bacterial species classification is successfully evaluated in a taxonomic framework. Although the proposed approach does not improve the overall accuracy compared to flat multi-class classification, it has some distinct advantages. First, it has better capabilities for distinguishing species on which flat multi-class classification fails. Secondly, the hierarchical classification structure allows to easily evaluate and visualize the resolution of FAME data for the discrimination of bacterial species. Summarized, by phylogenetic learning we are able to situate and evaluate FAME-based bacterial species classification in a more informative context.

## Background

Chromatographic fatty acid methyl ester (FAME) profiling is used in many laboratories for bacterial identification. The fatty acid composition of bacterial species is genetically conserved and the measured composition is stable, when highly standardized culture, extraction and analytical conditions are used. More than 300 fatty acids have already been found in bacteria. Differences in chain length, positions of double bonds and the binding of functional groups make them very useful taxonomic markers [1,2]. In the last decades, FAME profiling has become a routine method since it is cheap, fast, automated and high-throughput. As a result, many institutes have set up private FAME databases to store the massively generated numbers of FAME profiles and, recently, a publicly accessible FAME database has been realized by some of the present authors [3]. Such databases are an ideal target for data mining and knowledge discovery. Where bacterial species identification is usually performed by comparing FAME profiles against identification libraries with fixed peak percentages, FAME-based bacterial species identification can be improved by the application of machine learning techniques [4,5]. However, different numerical studies on the resolution of FAME analysis for species discrimination have underscored that FAME profiling cannot be used to discriminate all species from one another [6-9]. Nevertheless, machine learning techniques for multi-class classification are able to maximally exploit the pattern information in the FAME data to delineate the different species that constitute the different classes in this multi-class classification problem [5].

At present, the gold standard for bacterial species discrimination is a DNA-DNA hybridization (DDH) percentage of 70%. Nonetheless, DDH should be performed in a polyphasic study of the species because phenotypic characteristics should agree with this definition [10,11]. Importantly, with the advent of 16S rRNA gene sequence analysis, Stackebrandt and Goebel [12] showed that species having 70% or greater DNA similarity usually correspond to a 16S rRNA gene sequence identity greater than 97%. Furthermore, Konstantinidis and co-workers evaluated the species definition in the perspective of whole-genome sequence analysis and showed that the 70% DDH standard correlates with a 95% average nucleotide identity [13,14]. Even though DNA reassociation is the gold standard for circumscribing the taxonomic rank of species and genome studies flourish, 16S rRNA gene sequence analysis is still widely preferred for species delineation for two important reasons: 16S rRNA gene sequence identity greater than 97% may indicate a specific species and sequencing the 16S rRNA gene has become much cheaper and faster due to technological advances. For these reasons, we also focus on the 16S rRNA gene in this work. It is, however, important to remark that, as a consequence of this explosive trend of gene sequencing, deposits in the public nucleotide sequence databases have witnessed an exponential growth. Nonetheless, sequence analysis and phylogenetic reconstruction studies should rely on high quality nucleotide sequences. With the exponential growth of the sequence databases, the number of poor quality sequences also grows extensively and sequence curation becomes indispensable. To circumvent manual curation, the SILVA database project allows users to retrieve quality controlled and aligned rRNA sequences as stored in the EMBL sequence database [15]. In relation to this work, we tackle the bacterial species classification problem by combining the information represented by aligned 16S rRNA gene SILVA sequences and FAME profiles. Due to technological advances, both these types of data can be easily obtained at very low cost. However, when used alone, FAME data has a limited ability to discriminate among species. Combining the knowledge contained in FAME profiles and 16S rRNA gene sequences could overcome some of these limitations.

At present, machine learning papers describing multi-class classification with classes structured in a taxonomy, or thus a tree topology, mainly focus on the area of web-, document-, text- and ontology-based classification. Many research problems involve multi-furcating tree nodes, and most papers deal with data instances primarily corresponding to multiple classes structured in this kind of hierarchical setting. Classification problems related to this issue are better known as multi-label classification. In machine learning terms, learning by exploiting hierarchical structure information is called hierarchical classification [16-23], learning with taxonomies [24] and structured label learning [25]. However, these studies do not explicitly involve hierarchical classification for single-label multi-class classification, meaning that each data instance is classified at leaf level. From another perspective, hierarchical classification has also been proposed for standard multi-class classification tasks. In this setting, the idea consists of improving multi-class classification methods by constructing a tree of binary classifiers [26-28]. The tree architecture is defined by the considered data and tree inference is based on different algorithms for distance calculation between the considered classes.

In contrast to previous work, where typically a single type of data was used for bacterial species identification, we evaluate the integration of taxonomic and phylogenetic knowledge into FAME-based classification models. To this end, species of the genus *Bacillus *are considered. We design supervised machine learning techniques to automatically discriminate FAME profiles of bacteria at species level, in a hierarchical classification setting where the labels correspond to the different species. In particular, clustering methods define the taxonomic or phylogenic tree in a first stage and Random Forest (RF) classifiers are trained on FAME profiles in these trees in a second stage. Two different strategies for the integration of taxonomic and phylogenetic knowledge are investigated. As a proof-of-concept, we consider the integration of relationships between species solely based on FAME data. Herein, a FAME tree is constructed by divisive clustering and evaluated for hierarchical multi-class classification. In the core part of this paper, we consider knowledge integration from the perspective of bacterial phylogeny. Using 16S rRNA gene sequence analysis, phylogenetic trees are constructed and subsequently used for hierarchical single-label multi-class classification, in which FAME data serve as input. This last strategy is further referred to as phylogenetic learning, an approach that combines two types of data: 16S rRNA gene data is considered to incorporate phylogenetic knowledge in the form of a hierarchy or tree and the hierarchically ordered classifiers are constructed based on FAME data. Our tests indicate that this new approach resolves some of the classification tasks that classifiers only based on FAME data could not achieve. In relation to other work, the use of phylogenetic tree information has already been considered for classification of protein-protein interaction [29] and multi-class classification in a taxonomic context has already been performed based on genomic sequence data [30,31]. However, the incorporation of phylogenetic information in hierarchical classification models for bacterial species has not been investigated so far.

## Results and Discussion

Because FAME data does not allow for a global discrimination of bacterial species, we tackle the bacterial species classification problem by combining FAME data with taxonomic or phylogenetic knowledge. Therefore, within the framework of bacterial taxonomy, an interesting direction for subsequent machine learning research is that of integrating this knowledge. This is easily achieved by learning in a hierarchical scheme or an inferred tree. Two approaches are considered: tree inference by FAME data and inference of phylogenetic trees based on 16S rRNA gene sequences. In this paper, we evaluate the integration of these two particular types of knowledge into the FAME-based bacterial species classification problem.

### Learning taxonomies

**Figure 1:**
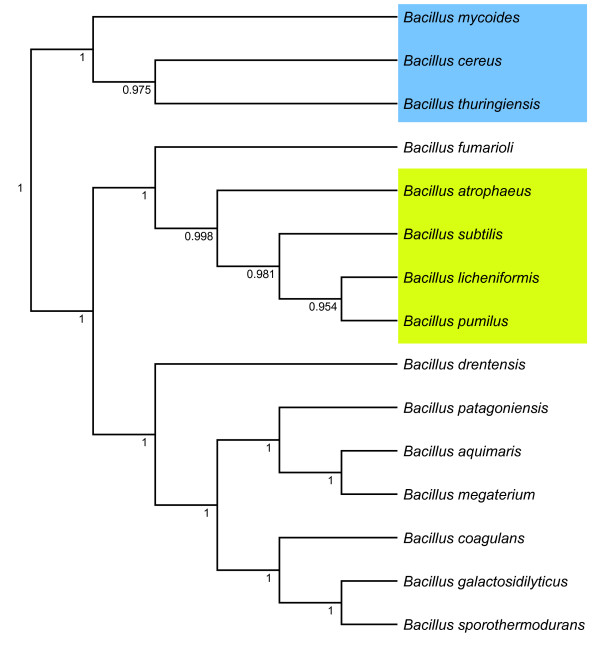
**Divisive clustering tree**. Phylogenetic tree resulting from the divisive clustering of the FAME data of 15 *Bacillus *species based on classification by Random Forests. Clustering is based on AUC and average linkage of the probability estimates calculated from identification by Random Forests. At the different nodes the corresponding AUC value is reported. The *Bacillus cereus *and *Bacillus subtilis *groups are coloured in blue and green, respectively.

As a first step, we investigated the possibility of reconstructing a small part of the phylogenetic structure of the genus *Bacillus *by FAME data and RFs. Divisive clustering with classifier performance as splitting criterion gives rise to a particular tree. In this tree, the different species are hierarchically ordered by similarities in the FAME data. In the resulting top-down approach, all possible splits between species or classes are initially considered in the root node and, subsequently, the split corresponding to the highest RF accuracy is chosen. Recursively, the same splitting procedure is performed on the corresponding subsets of the initial data set. Since the data set consists of a small number of FAME profiles for the majority of species, we preferred to use a divisive clustering algorithm over an agglomerative clustering algorithm. The latter approach has as disadvantage that it builds a tree in a bottom-up manner, so that, in our setting, the clustering at leaf level could be obtained from the results of unreliable classifiers (due to a small number of FAME profiles for many species). Conversely, we chose to work top-down with a divisive clustering algorithm, because we wanted to avoid this type of instability in the tree construction phase. Initially, we performed a proof-of-concept experiment based on a small data set of 15 species, as selected from the original data set. Only species corresponding to a large number (at least 11) of FAME profiles were selected. About half of the selected species belong to the two known *Bacillus *species groups, the *Bacillus cereus *group and the *Bacillus subtilis *group. Hierarchical divisive clustering starts in the root node with the training and evaluation of 16383 RF classifiers. In subsequent steps of the clustering algorithm, classifier training becomes less time-consuming, because the number of trained classifiers decreases exponentially for the remaining subtrees. In the end, a total number of 18589 classifiers were trained. The computing time to build and evaluate the complete species hierarchy was 65 h 10 m 22 s. By this initial experiment, we evaluated whether a FAME tree constructed with divisive clustering indeed reveals the relations between the species of the different species groups. Figure 1 shows the resulting tree, in which no branch lengths are specified and AUC values of the RF classifiers are given at each internal node. The species representing the *Bacillus cereus *group or the *Bacillus subtilis *group are clearly clustered together under the same parent nodes. The two groups are coloured in blue and green, respectively.

Consequently, one can conclude that FAME data allows to discriminate between both species groups. We did not expect such a result because of the large number of combinations and the high similarities between the FAME profiles. However, this experiment clearly shows that RFs take advantage of the relatedness between species and/or groups of species. Consequently, building a FAME tree using classification techniques as treeing method could be a good base for further knowledge integration. Therefore, we evaluated the tree constructed from the different RF models also as a hierarchical classification scheme. This classification task follows the main strategy as reported by [26-28]. Subsequent to the construction of the tree, a RF classifier is retrained at each node of the tree, so that now the different classifier parameters are optimized by a grid search, as mentioned in the subsection Divisive Clustering. To this end, we considered both 3-fold and 11-fold stratified cross-validation.

The corresponding results are reported in the upper part of Table 1. These results show that hierarchical single-label multi-class classification with 3-fold stratified cross-validation performs slightly worse than flat multi-class classification (see bottom part of Table 1). Performing 11-fold stratified cross-validation, however, results in a slightly better performance than flat multi-class classification. In summary, for the 15 species data set, we can conclude that hierarchical single-label multi-class classification results in a performance comparable to that obtained with flat multi-class classification. Nonetheless, we are mainly interested in the classification of the 74 *Bacillus *species present in our data set. Upscaling this experiment from 15 classes to 74 is, however, computationally infeasible, because the number of classifiers to be trained increases exponentially with the number of classes. When considering these 74 classes in our FAME data set, 2^73 ^- 1 classifiers must be trained in the root node. This cannot be realized in a reasonable computing time, even when multiple processors are used in parallel. Furthermore, to obtain a good classification performance in the proof-of-concept experiment, we only selected species represented by a reasonable amount of data. Nonetheless, in the full data set, a lot of classes are present with a small number of FAME profiles (e.g. only 3 or 4 profiles) which may result in an unreliable FAME tree. Even though the results of this experiment with 15 species are promising, for the reasons above, we did not further consider knowledge integration by divisive clustering of FAME profiles.

**Table 1:** Results from the hierarchical single-label multi-class classification, phylogenetic learning and flat multi-class classification experiments.

Classification Results						
	**AUC**	**Sensitivity**	**Precision**	**NaN**	**F-score**	**NaN**

**HSMC - 15 species**						
HSMC		0.887 ± 0.214	0.945 ± 0.059	0	0.895 ± 0.179	0
HSMC (11-fold CV)		0.916 ± 0.130	0.956 ± 0.037	0	0.930 ± 0.083	0

**PhyLearn - 15 species**						
PhyLearn - NJ		0.992 ± 0.007	0.954 ± 0.041	0	0.924 ± 0.099	0
PhyLearn - UPGMA		0.860 ± 0.211	0.931 ± 0.064	0	0.873 ± 0.153	0

**PhyLearn - 74 species**						
PhyLearn - NJ		0.741 ± 0.237	0.846 ± 0.181	1	0.768 ± 0.181	1
PhyLearn - UPGMA		0.684 ± 0.256	0.860 ± 0.174	2	0.741 ± 0.180	2

**Multi-class**						
15 species	0.992 ± 0.010	0.902 ± 0.170	0.944 ± 0.054	0	0.911 ± 0.124	0
74 species	0.982 ± 0.042	0.851 ± 0.189	0.901 ± 0.121	0	0.863 ± 0.145	0

In this table, the three strategies are abbreviated as 'HSMC', 'PhyLearn' and 'Multi-class', respectively. The results of these three strategies are reported in the upper, middle and bottom part of the table, respectively. The results of hierarchical single-label multi-class classification are based on the FAME tree resulting from the divisive clustering experiment. Only the 15 species data set was considered and 3-fold and 11-fold stratified cross-validation (CV) was performed. In the case of phylogenetic learning, two 16S rRNA gene trees were used as template: neighbor-joining (NJ) and unweighted pair group method with arithmetic mean (UPGMA). For PhyLearn, both the 15 and the 74 species data set were considered and all PhyLearn experiments were performed using 3-fold stratified CV. Also the flat multi-class experiments were validated by this CV strategy. In the three strategies, classification performance was evaluated based on the pooled test set. Metrics reported are the area under the ROC curve (AUC), sensitivity, precision and F-score. Based on a multi-class confusion matrix, statistics were calculated in a one-versus-other setting with averaging of the corresponding statistic over the different classes. Standard deviations are also reported. NaN denotes the number of classes that have resulted in a value ∞ (only in case of precision and F-score).

### Phylogenetic learning

An alternative to the construction of a FAME tree is to infer a tree on data with a good resolution for species discrimination. In this perspective, the best possibilities are DDH, whole-genome sequence analysis and multi-locus sequence analysis (MLSA). The lack of a sufficient amount of high-quality data, however, makes these techniques not very attractive. Therefore, yet another alternative is to focus on 16S rRNA gene sequencing. This technique is widely preferred for species delineation because of improved sequencing technology and the availability of public sequence databases. Nonetheless, the 16S rRNA gene may not allow for a delineation of every species [10,12-14]. Currently, 16S rRNA gene sequence analysis is one of the techniques widely used in microbiology for phylogenetic analysis. We integrated this knowledge in FAME-based bacterial species classification models to evaluate species identification as well as the resolution of FAME analysis for species discrimination within a phylogenetic framework.

When using this technique as a starting point for knowledge integration, high quality 16S rRNA gene sequences can be exported from the SILVA database. This database subjects EMBL 16S rRNA gene sequences to different control procedures and annotates the corresponding sequences with quality scores [15]. In this way, we selected exactly one 16S rRNA gene sequence for each type strain of each *Bacillus *species present in the original FAME data set. Note that the type strain of a bacterial species is the fixed name bearer of the species (according to the bacterial code [32]) and its phylogenetic position is hence determinative in the taxonomic framework.

After sequence selection, distance matrices were calculated using the Jukes-Cantor nucleotide evolution model and two phylogenetic trees were constructed accordingly, respectively with the neighbor joining method (NJ) and the unweighted pair group method with arithmetic mean (UPGMA) [33-37]. The respective trees are shown in Additional files 1 and 2. Subsequently, we used these binary trees as templates for hierarchical FAME-based species classification. As this hierarchical classification relies on a phylogenetic tree, we call this approach *phylogenetic learning*. As the binary tree classifier is based on a rooted tree structure, we initially selected the NJ and UPGMA methods as these basically infer rooted trees, even though several other tree inference methods exist (e.g. maximum parsimony and maximum likelihood) [36]. Two different methods were considered in order to allow for a comparison of binary tree classifiers based on different trees.

The constructed RF classifiers were evaluated for distinguishing between the FAME patterns of the two underlying groups of species in every node of the tree. The collection of binary classifiers should be regarded as one classifier wrapping the multiple hierarchically structured classifiers. Three-fold stratified cross-validation for error estimation was performed during the training process of each classifier with pooling of the test results of all folds [38,39], i.e. the predictions on test data are pooled together in one big set, and the performance measures are calculated on this set. The results of phylogenetic learning based on the NJ and UPGMA trees are reported in the middle part of Table 1. These results are compared with those obtained from a FAME-based flat multi-class classification (see bottom part of Table 1), where only one multi-class classifier is trained by the same cross-validation strategy. First of all, we also evaluated phylogenetic learning and flat multi-class classification for the 15 species data set (as selected in the previous subsection). The corresponding results are reported in Table 1. Note that the flat multi-class classification with 3-fold stratified cross-validation in this study differs from the flat multi-class classification strategy performed in [5]. In the latter study, 10 repeated experiments were carried out with averaging of the classifier performance on a randomly sampled test set. Average AUC, sensitivity and precision were then given by 0.988, 0.847 and 0.908, respectively. These metric values are approximately equal to the values obtained in the present study. As a result, the cross-validation with pooled metric calculation in a flat multi-class setting does not lead to very different estimates of classification performance, when compared to the random test set selection carried out in [5].

Even though flat multi-class classification of the 15 species data set results in a very high AUC value of 0.992, it is also interesting to see that higher sensitivity and F-score values are obtained by phylogenetic learning on this data set (based on the NJ tree). Conversely, phylogenetic learning based on a UPGMA tree performs slightly worse than flat multi-class classification. When the study is scaled up to 74 species, flat multi-class classification performs better than phylogenetic learning on both trees. For the NJ and UPGMA trees, the difference in sensitivity between both techniques and flat multi-class classification is 11% and 16.7%, respectively, while the difference in F-score is, respectively, 9.5% and 12.2%. The contrast between the two data sets is, logically, based on the larger number of relations between the different species and the more complex hierarchical structure of the data. The main reason for the lower prediction performance of phylogenetic learning can be attributed to the 16S rRNA gene phylogenetic trees that define the multiple learning tasks. These could become quite hard to solve when classifying the species based on FAME data. Flat multi-class classification is not confronted with these restrictions at all and allows for more flexible solutions. Moreover, in a 74 species hierarchical learning system, the probability of a misclassification along the identification path in the tree is much larger than the misclassification probability in a 15 species hierarchy. Also, in the 74 species data set, some species are known to be very closely related to each other, increasing the probability of misclassification in the hierarchy. Despite a lower classification performance compared to flat multi-class classification, phylogenetic learning allows to evaluate the classification scheme at node level. In this way, it is possible to analyze the resolution of FAME data at different tree levels. Ultimately, the goal of this approach will be to investigate how a particular pruning strategy could be applied by which those species will be grouped that are hard to classify by the machine learning method of interest. As a consequence, it will also become possible to report identification scores for groups of species that are very related in their FAME content.

Further investigation could also be done on the improvement of classification performance. For instance, a variable misclassification cost could be defined along the classification path. As an example, nodes splitting groups of classes could be evaluated differently than nodes splitting one species from a group of classes and splitting two leaves. In the latter case, a more severe misclassification cost can be assigned. Another approach could account for the different branch lengths of the phylogenetic tree.

As the multi-class classification problem is tackled by hierarchically structured binary classifiers, it is also interesting to look at the individual classes. As mentioned in the section Methods, a multi-class confusion matrix is generated by classification of each test profile and counting the different types of errors that are made. As such, this matrix is constructed from several two-class confusion matrices in a one-versus-all manner, in which, for each class, the positive class is the class under consideration, while the negative class corresponds to all other classes. Using the iTol webtool [40], we have plotted a bar diagram of sensitivity and F-score values along the tree and have aligned the corresponding bars with the corresponding leaf or class of the tree. When an F-score resulted in a value of ∞ (i.e. sensitivity and precision equal zero), no bar is visualized. In this way, rapid inspection is possible to detect those classes that are hard to identify by the phylogenetic learning model and the flat multi-class classifier.

**Figure 2:**
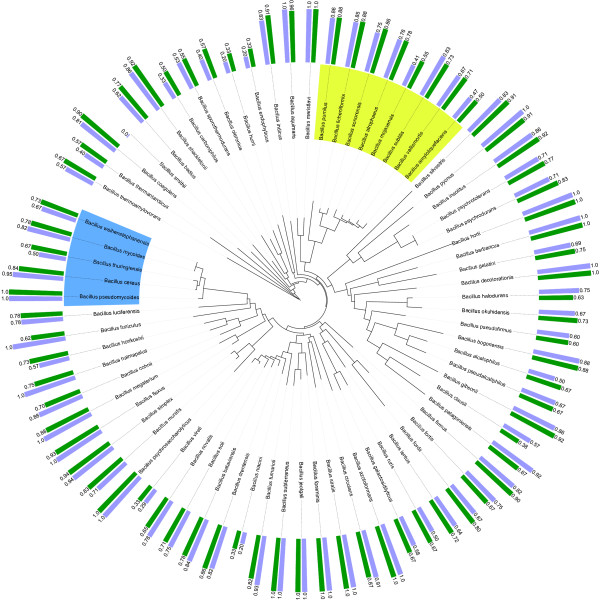
**Sensitivity and F-score values by phylogenetic learning based on a 16S rRNA gene NJ tree**. For each *Bacillus *species, the corresponding sensitivity and F-score value of phylogenetic learning based on a 16S rRNA gene NJ tree is displayed. Sensitivity is indicated by the light blue bars, F-score by the green bars. The tree is visualized using the iTol webtool [40]. The *Bacillus cereus *and *Bacillus subtilis *groups are coloured in blue and green, respectively.

**Figure 3:**
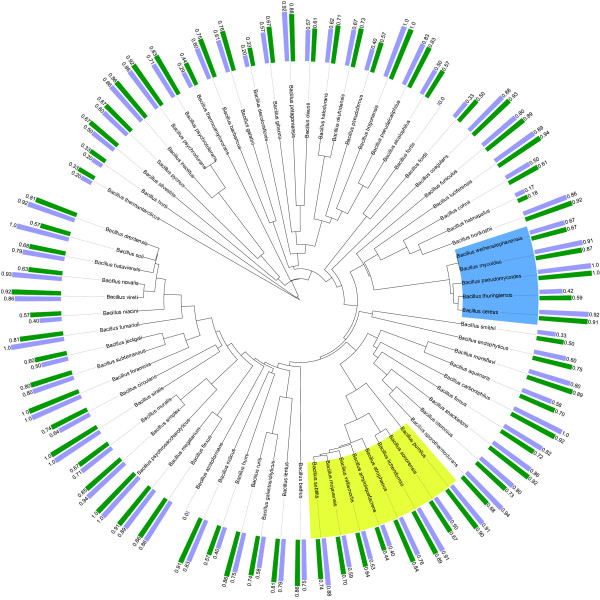
**Sensitivity and F-score values by phylogenetic learning based on a 16S rRNA gene UPGMA tree**. For each *Bacillus *species, the corresponding sensitivity and F-score value of phylogenetic learning based on a 16S rRNA gene UPGMA tree is displayed. Sensitivity is indicated by the light blue bars, F-score by the green bars. The tree is visualized using the iTol webtool [40]. The *Bacillus cereus *and *Bacillus subtilis *groups are coloured in blue and green, respectively.

**Figure 4:**
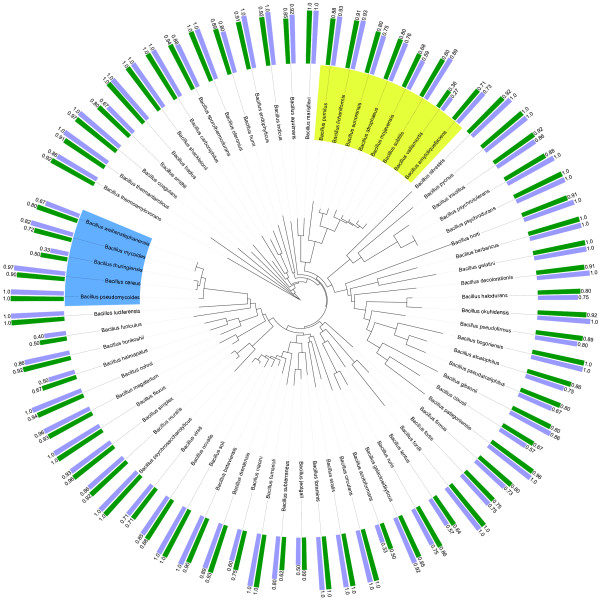
**Sensitivity and F-score values for flat multi-class classification**. For each *Bacillus *species, the corresponding sensitivity and F-score value of flat multi-class classification is displayed along the 16S rRNA gene NJ tree. Sensitivity is indicated by the light blue bars, F-score by the green bars. The tree is visualized using the iTol webtool [40]. The *Bacillus cereus *and *Bacillus subtilis *groups are coloured in blue and green, respectively.

**Figure 5:**
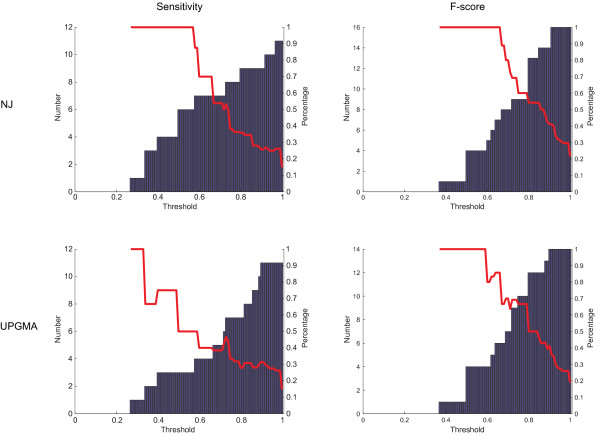
**Comparison of performance at class level**. For each class, sensitivity and F-score values resulting from phylogenetic learning based on a 16S rRNA gene NJ or UPGMA tree are compared to those obtained by flat multi-class classification. Four plots are given. The X-axis corresponds to thresholds set on the corresponding metric values. Threshold steps of 0.01 are chosen. For each threshold, flat multi-class classification is evaluated at class level and those classes with metric values smaller than or equal to the threshold are selected. Classification performance by phylogenetic learning is analyzed at class level for each set of classes. The Y-axis on the left projects the number of phylogenetic learning classes that have a higher metric value than those obtained by flat multi-class classification. The red line expresses this number, relative to the size of the corresponding set (Y-axis on the right).

The results of phylogenetic learning with NJ and UPGMA trees and those of flat multi-class classification are displayed in Figures 2, 3 and 4. In case of flat multi-class classification, the metric values are displayed along the 16S rRNA gene NJ tree. When comparing the sensitivity values of each species obtained by phylogenetic learning based on the two considered 16S rRNA gene trees to those obtained by multi-class classification, only 15% of the species have a higher sensitivity value. 57% and 61% of the species have a lower sensitivity value, for the NJ and the UPGMA tree, respectively. In case of the F-score, 22% and 19% of the species have a higher F-score value, while 69% and 70% of the species have a lower sensitivity value, for both trees respectively. Nonetheless, when looking more deeply into the results, those classes that are hard to distinguish from the other classes in a multi-class classification setting are better identified in the hiearchical classification setting. This is clearly illustrated by the cumulative plot in Figure 5. In this figure, identification by phylogenetic learning is compared to flat multi-class identification at class level. Even though phylogenetic learning performs globally worse than flat multi-class classification, it is clear that, when considering a threshold of 0.5-0.6, phylogenetic learning has an added value due to better identification of classes that are not well identified by multi-class classification.

**Figure 6:**
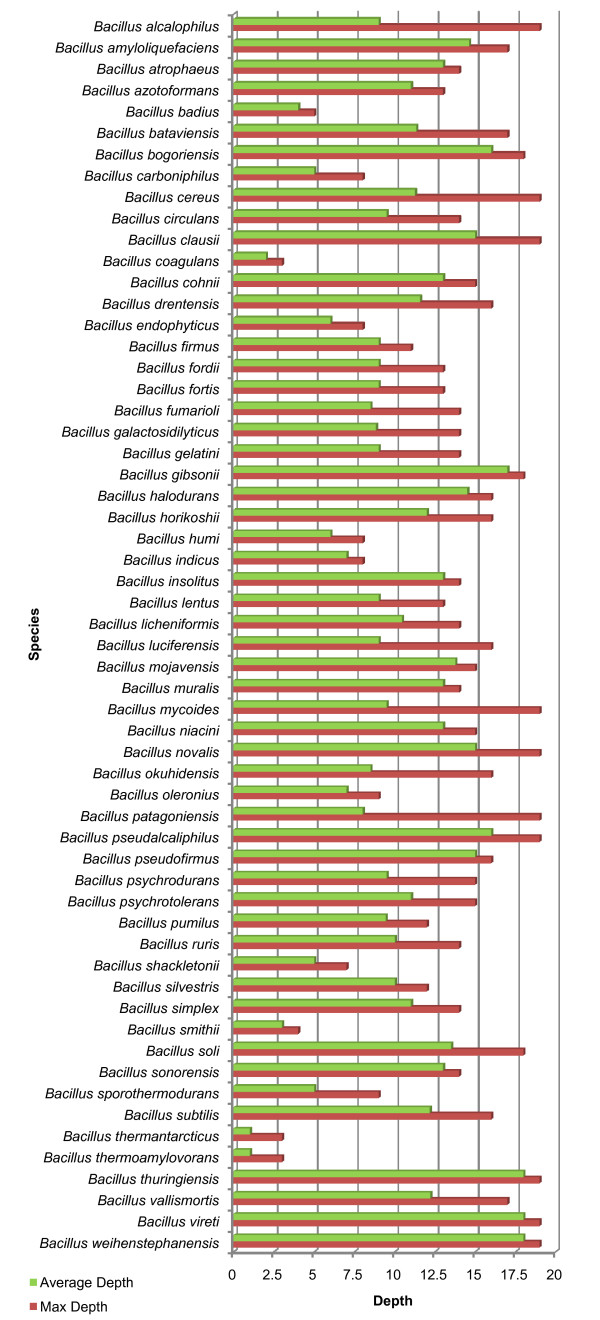
**Average misclassification depth of phylogenetic learning based on a 16S rRNA gene NJ tree**. The average depth of the misclassified test profiles of each species is visualized for phylogenetic learning based on a 16S rRNA gene NJ tree. Depth equals the number of nodes along the classification path until misclassification occurs (the corresponding node also included) and corresponds to the green bars. The maximum or correct depth is shown by the red bars. Maximum depth equals the number of nodes along the true phylogenetic path (leaf included).

As mentioned above, a hierarchical classification structure allows to analyze where misclassification occurs in the tree. This offers new possibilities to further analyze the resolution of FAME data for species discrimination. Furthermore, it is also interesting to calculate an average misclassification path length. The results for phylogenetic learning based on the NJ tree are visualized in Figure 6. The results for phylogenetic learning based on the UPGMA tree are similar and are visualized in Additional file 3. Herein, importantly, we only considered misclassified test profiles. It becomes clear from both figures that misclassification mostly occurs at nodes near the correct leaf. This is not very surprising as, based on FAME data, a lot of species cannot be distinguished from each other. This again shows that the resolution of FAME analysis is restricted to distantly related species and species groups.

## Conclusions

In this work, we combined FAME data and 16S rRNA gene sequences in bacterial species classification models. Supervised machine learning techniques have shown their value in the past for distinguishing FAME profiles of different *Bacillus *species. However, bacterial species can be closely related in terms of FAME content, making it hard to achieve a high classification performance. With this study, we approached the classification problem from a taxonomic perspective, in which a hierarchical classification scheme with binary tree classifiers was adopted. This machine learning technique is a perfect method for the integration of taxonomic relationships between the different classes or species.

Two strategies were followed with regard to tree inference. First, a FAME tree was constructed by divisive clustering, in which classifier performance was considered as splitting criterion. In this setting, one can see knowledge integration as the inclusion of similarities in the FAME profiles of the considered species. Due to a computational bottleneck, this approach was restricted to a proof-of-concept experiment with a data set of 15 species. Relatively good results were obtained, as closely related species could be retained out of the massive amount of computed clusters. Furthermore, for these 15 species, we observed a good classification performance that is comparable to flat multi-class classification.

As a second strategy, we considered knowledge integration based on 16S rRNA gene data. Using quality controlled 16S rRNA gene sequences, two rooted phylogenetic trees were constructed for the type strains of 74 *Bacillus *species. The two trees, respectively constructed with the NJ and UPGMA treeing methods, subsequently served as taxonomies for a hierarchical classification scheme with base classifiers trained on FAME data at each node. Due to the integration of 16S rRNA gene as a phylogenetic marker, we called this approach phylogenetic learning. The main advantage of phylogenetic learning, when compared to flat multi-class classification, lies in the exploitation of the taxonomic relationships, so that the results can be easily visualized. Moreover, the results can be better interpreted in a post-processing phase, for example by pruning the classification tree based on the classifier performance along the tree. Given the limitations of FAME data for species discrimination, pruning allows to restrict identification to internal nodes and put the identification results in a taxonomic context. And, herein, a hierarchical classification scheme is a perfect choice, contrary to flat multi-class classification models, which cannot easily exploit taxonomic relationships, and do not produce a score of separability or identification of a certain species, as compared to (closely) related species.

Furthermore, when evaluating the identification performance of phylogenetic learning at leaf level, another clear advantage is seen. Due to the hierarchical class structure, the phylogenetic learning approach improves the identification for species that are incorrectly classified by flat multi-class classification. So, in some sense, the method also results in a better performance, although we have to admit that phylogenetic learning with the NJ and UPGMA trees shows to be less accurate than flat multi-class classification, when evaluating the method as a global classification scheme. Yet, as explained above, this was not the goal of this study.

## Methods

### Fatty Acid Methyl Ester data

The *Bacillus *fatty acid methyl ester (FAME) data set of Slabbinck *et al*. [5] was used. Basically, gas chromatographic FAME profiles were generated after growing the bacteria, as described in the protocol of the Sherlock Microbial Identification System of MIDI Inc. (Newark, DE, USA). This protocol defines a standard for growth and culturing of bacterial strains and reproducibility and interpretability of the profiles is only possible by working under the described conditions. Specifically, this protocol recommends 24 h of growth on trypticase soy broth agar at a temperature of 28°C. Subsequent to gas chromatographic analysis, the use of a calibration mix and the TSBA 50 peak naming table, the resulting FAME profiles are standardized by calculating relative peak areas for each named peak with respect to the total named area. Our data set covers 71 identified fatty acids (or features) and 74 validly published *Bacillus *species (or classes). This number is about one half of the *Bacillus *species published by IJSEM as of March 2008 [41]. The total number of FAME profiles (or data instances) is 961. The resulting data set was exported from the joint FAME database of both the Laboratory of Microbiology (Ghent University, Belgium) and the BCCM™/LMG Bacteria collection.

### Random Forests

In 2001, Breiman [42] proposed a new machine learning technique consisting of an ensemble of classification trees, better known as Random Forests. A Random Forest (RF) classifier can be defined as a classifier consisting of a collection of decision trees, where each tree casts a unit vote for the most popular class at each input. Each tree in the forest is first grown using *N *training data points that are randomly sampled from the original data set, and subsequently evaluated by the remaining test data points. *N *equals about two-thirds of the size of the original data set. Importantly, the random sampling procedure is done with replacement, better known as bootstrapping. Aggregation of the classifiers built upon the bootstrap samples is called bagging or bootstrap aggregation. Bagging leads to a specific data set and the remaining data points are called 'out-of-bag'. When using the latter data set for evaluating the accuracy of the grown tree, the prediction error is, therefore, called the out-of-bag error. Randomly sampling data sets to grow the different trees of the forest corresponds to one of the two randomness factors of RFs. The second factor lies in the random split selection. When *M *features are present in the original data set, *m *features (*m *<<*M*) are sampled randomly out of *M *features to split each node of the tree. The final split is determined by the best split based on the *m *randomly sampled features [42,43]. Note that *m *is held constant during the growth process of the forest. Each tree is maximally extended and no pruning occurs. In our study, a grid search was performed to optimize the number of trees and the number of split variables. All numbers of features were considered for split variable selection and 1000 to 4000 trees in steps of 250 trees were selected for tuning the number of trees.

Evaluation of the classification accuracy of a RF classifier is based on the different out-of-bag data sets. For each data point *i *of the data set, all out-of-bag data sets containing *i *are considered. Data point *i *is put down the trees corresponding to the respective out-of-bag data sets. Class *j *is set to be the class that got most votes every time data point *i *was out-of-bag. The proportion of times that *j *is not equal to the true class averaged over all data points corresponds to the out-of-bag error estimate. A RF classifier consisting of low-correlated trees with a high individual strength results in optimal generalization and a high accuracy. Moreover, from the law of large numbers and the tree structure, it follows that the generalization error of RFs converges to a certain value, implying that a RF classifier does not overfit, given a large number of trees in the forest [42]. However, Hastie et al. [44] remark that changing the number of trees in the forest does not cause overfitting, given that not all features are used as split variables and the number of noise variables is reduced.

Modeling was performed by the RFs software available at the website of Leo Breiman [43].

### Divisive clustering

A divisive clustering algorithm builds a top-down cluster hierarchy or tree, starting from a single cluster that corresponds to the entire dataset (i.e. the root of the tree). In every iteration of the algorithm, one cluster is selected and split into two new clusters. If the distances between all points in the clusters are considered, then this results in a traditional unsupervised clustering procedure. Conversely, supervised divisive clustering also takes the class labels of the respective data points into account, and calculates only distances between the data points with differing class labels. As a consequence, the final number of clusters in supervised clustering equals the number of classes present in the original data set [45,46].

Popular divisive clustering strategies are single linkage, complete linkage, average linkage, Ward linkage, etc. In these strategies, multiple metrics can be applied as distance measure. In our initial study, for the construction of a tree using solely FAME data, we calculated the distance metric based on the performance of binary classifiers that were trained for all possible splits of the data. In this setting, cluster distances are not simply computed from FAME profiles, but also the class labels are taken into account. As first splitting criterion, the area under the ROC curve (AUC) was considered, and in case of ties, the splitting was refined by accounting for the average linkage of the probability estimates of both classes. The rationale behind this last splitting criterion is that the classifier corresponding to the largest average distance between the probability estimates should be preferred over other classifiers. The Euclidean distance was considered as metric on the probability estimates.

For each level in the divisive top-down setting, a RF classifier is built for all possible two-group combinations of all considered species or classes. As such, all combinations correspond to a two-class classification task. For each node or level, this results in 2^*n*-1 ^- 1 combinations, with *n *the number of classes considered. Note that, when considering four classes, the combination of classes 1 and 2 automatically excludes the combination of classes 3 and 4. The divisive clustering stops when only two-class clusters are retained. To speed up the divisive clustering and classification process, no grid search and no cross-validation were considered. Specifically, the initial FAME data set was randomly splitted in a stratified train and test set. One-third of the data set was used for testing. For each combination, the according profiles for training and testing were sampled from these two subsets. The forest size was optimized using the default number of split variables (*m *= , with *M *the number of features). After fixing the forest size resulting in the lowest error rate, the optimal number of split variables was selected among , 2*M *and , again corresponding to the lowest error rate. Ultimately, a rooted tree was constructed with equal branch lengths and the different nodes were labeled with the corresponding AUC value. The resulting tree was visualized with the treeing method of the TaxonGap software [47].

As an initial proof-of-concept, 15 *Bacillus *species were selected from the original data set. Selection was based on classes with reasonable sample size and classes that are taxonomically closely related to each other, e.g. species of the *Bacillus cereus *and *Bacillus subtilis *groups. The first selection criterion was chosen to avoid heavily imbalanced data subsets. The following species with respective number of profiles were selected: *Bacillus aquimaris *(12), Bacillus atrophaeus^*s ^(21), Bacillus cereus^*c ^(62), *Bacillus coagulans *(32), *Bacillus drentensis *(38), *Bacillus fumarioli *(28), *Bacillus galactosidilyticus *(12), Bacillus licheniformis^*s ^(74), *Bacillus megaterium *(28), Bacillus mycoides^*c ^(11), *Bacillus patagoniensis *(12), Bacillus pumilus^*s ^(57), *Bacillus sporothermodurans *(17), Bacillus subtilis^*s ^(64) and Bacillus thuringiensis^*c ^(12). Species annotated with '^*c^' belong to the *Bacillus cereus *group, while species annotated with '^*s^' belong to a species of the *Bacillus subtilis *group [41,48,49]. It is expected that the species of these two groups cluster together.

To further speed up the clustering process, computations were performed in parallel on an Intel Blade cluster.

### Phylogenetic analysis

The SILVA database was used for 16S rRNA gene sequence selection. SILVA provides quality checked and aligned 16S rRNA gene sequences. For each type strain of each species present in our data set, a 16S rRNA gene sequence was selected. If multiple 16S rRNA gene sequences for each type strain were available, selection of the final sequence was based on best quality and longest sequence length. In SILVA, quality is denoted three-fold: pintail quality for sequence anomaly detection, sequence quality and aligment quality [15]. A list of the selected accession numbers can be found in Additional file 4.

Sequence distance calculation was performed by PHYLIP, the PHYLogeny Inference Package version 3.68, using the program Dnadist [35,36]. The Jukes Cantor evolution model was used for correcting the nucleotide distances. This DNA sequence evolution model assumes an equal and independent change rate for each nucleotide. So, substitution of one nucleotide by one of the three other nucleotides occurs with equal probability. All other parameters were used as default except for the input format. Based on the resulting distance matrix, a NJ and a UPGMA tree was created using the PHYLIP program Neighbor [33,34,37]. Default parameter settings were used, except for a randomized input order of the species. Phylogenetic trees were created for the species present in both the full data set and in the 15 species data set. All trees are visualized with the iTol webtool version 1.5 [40]

### Phylogenetic learning

Based on the 16S rRNA gene phylogenetic trees, a classification scheme with a hierarchical class structure was developed. As such, a rooted phylogenetic tree can be regarded as a directed acyclic graph with bifurcating nodes. The main idea is similar to that of binary tree classifiers [26-28]. However, in contrast to our study, these authors inferred a tree from the data used for classification, while we considered phylogenetic information (16S rRNA gene) for tree inference and used FAME data solely for classification. We call this approach phylogenetic learning. As a simple, naïve approach, at each node of the 16S rRNA gene phylogenetic tree, a two-class RF classifier was trained, based on a subset of the FAME data set. Herein, only the subset of profiles belonging to that part of the tree was taken for training and testing. The two branches of the node defined the two groups of the binary classification task, and at each node, a positive and negative dummy class label was created.

Given the tree hierarchy, classifiers constructed on terminal nodes and a certain number of parent nodes can become biased due to a small training set size. Herein, terminal nodes are regarded as nodes splitting into two leaves. As a consequence of the small sample size for certain species, splitting the data set into a training set and a test set was not an option. We simply overcame this issue by using cross-validation techniques, as explained in the next paragraph. Furthermore, the classification performance could easily be evaluated, since each profile was presented to the classification hierarchy and its path was fixed. In the case of an incorrectly classified profile at a specific node in the tree, propagation along the true path stopped, and the corresponding profile was further identified along the predicted path. Therefore, the path and ultimate predicted class of each profile could be determined and a multi-class confusion matrix could be generated for statistical analysis (discussed in the next subsection). As an interesting feature, this method offers the possibility to investigate where misclassification mostly occurs along the phylogenetic tree. Hence, the misclassification distance for each species could be estimated by averaging the correct path length of each incorrectly classified profile. This implies that, for such a profile, the correct path length was incremented each time the corresponding classifier resulted in identification of the true branch.

Incrementing continued until the considered profile was incorrectly classified. Note that the node resulting in misclassification also incremented the path length and that the path length was also incremented when ultimate identification occurred in the correct leaf. In the latter case, the correct path length equals the maximal path length. For each class, the average correct path length was plotted against the maximal path length. An example is visualized in Figure 6.

### Cross-validation and statistical analysis

In machine learning studies, data sets are typically split in a training set and a test set, where the latter contains mostly one-third to one-half of the data set. This test set is used as hold-out set for estimation of the final classifier error rate. To prevent over- and underfitting, parameter optimization during the training phase should not be performed on the test set, but on a separate validation set [45]. To overcome this problem, cross-validation can be used. Herein, the training set is split in *k *separate validation sets for which *k*-fold cross-validation should be performed. In each step of the cross-validation, a different validation set is used to retrieve the optimal parameter combination, tuned during training on the *k *- 1 other validation sets [45]. In relation to our problem setting, two important issues should be taken into account. Our data set is not particularly large and some classes correspond to a (very) small number of profiles. Due to the small class sizes present in our data set, we chose to use the test set also as validation set, even though it is still better experimental practice to perform a cross-validation on the training set for larger sample sizes. In case of our FAME data set, two additional problems arise: classes are imbalanced, meaning that a different number of profiles is present for each species, and as mentioned above, many species contain only a small number of profiles. To tackle a possible imbalance effect on the classifier performance, the true error rate can be estimated by stratifying train and test sets [38]. For the second case, classification will also become problematic when two-class classifiers are created based on small data sets. This can be solved by performing cross-validation for performance estimation [39]. A three-fold stratified cross-validation was performed for both the hierarchical classification and flat multi-class classification. To prevent overfitting, the number of folds is set equal to the minimum number of profiles over all bacterial species, which is three in our case. In this perspective, the stratification proportion equals one-third. Given the identical nature of the probability estimates resulting from each RF model, we chose to aggregate all test sets in a joint test set for performance evaluation. This method is also better known as pooling [50]. Finally, for the pooled test set, an average of the error estimate for each class in a one-versus-all setting was calculated, next to the average and standard deviation of the error estimates over all classes. Statistics calculated were the AUC, accuracy, sensitivity, precision and F-score.

Besides the calculation of global performance measures, the performance at class level between flat multi-class classification and phylogenetic learning was also compared. The comparison is visualized in a cumulative plot (see Figure 5). Initially, flat multi-class classification and the corresponding classification results of each class were considered. A threshold was set on a metric using steps of 0.01. As metric, sensitivity and F-score were further analyzed. The corresponding thresholds are plotted along the X-axis.

For each threshold, those classes were selected corresponding to sensitivity or F-score values smaller than or equal to the threshold. Secondly, for each threshold and, thus, for each selected set of classes, the corresponding metric values obtained by phylogenetic learning were evaluated. The number of phylogenetic learning metric values that were larger than the corresponding metric values resulting from flat multi-class classification are plotted against the Y-axis on the left. Also, this number is expressed as a percentage of the corresponding class set size. The corresponding percentages are plotted against the Y-axis on the right.

## List of abbreviations

AUC: Area under the ROC curve; DDH: DNA-DNA hybridization; FAME: Fatty acid methyl ester; MLSA: Multi-locus sequence analysis; NJ: Neighbor joining; RF: Random Forest; ROC: Receiver operating characteristic; UPGMA: Unweighted pair group method with arithmetic mean.

## Authors' contributions

BS carried out the full study and drafted the manuscript. WW, BDB and PD participated in the classifier development and statistical analysis. PDV participated in the microbiological background of the work. All authors read and approved the final manuscript.

## Supplementary Material

Additional file 1 **16S rRNA gene neighbor-joining tree**. *Bacillus *16S rRNA gene neighbor-joining tree as constructed by PHYLIP 3.68 and based on sequences selected from the SILVA database. Only the species present in the original data set are visualized. The tree is visualized using the iTol webtool [40]. The *Bacillus cereus *and *Bacillus subtilis *groups are coloured in blue and green, respectively. Click here for file

Additional file 2 **16S rRNA gene UPGMA tree**. *Bacillus *16S rRNA gene UPGMA tree as constructed by PHYLIP 3.68 and based on sequences selected from the SILVA database. Only the species present in the original data set are visualized. The tree is visualized using the iTol webtool [40]. The *Bacillus cereus *and *Bacillus subtilis *groups are coloured in blue and green, respectively. Click here for file

Additional file 3 **Average misclassification depth of phylogenetic learning based on a UPGMA tree**. The average depth of the misclassified test profiles of each species is visualized for phylogenetic learning based on a UPGMA tree. Depth equals the number of nodes along the classification path until misclassification occurs (the corresponding node included) and corresponds to the green bars. The maximum or correct depth is shown by the red bars. Maximum depth equals the number of nodes along the true phylogenetic path (leaf included). Click here for file

Additional file 4 **Table S1 -- Strain list and corresponding 16S rRNA gene accession numbers**. List of the 74 considered *Bacillus *species together with their type strain number and the accession number of the selected 16S rRNA gene sequence. Click here for file
