# Supplementary figures and images for: From learning taxonomies to phylogenetic learning: Integration of 16S rRNA gene data into FAME-based bacterial classification
Source: BMC Bioinformatics. 2010 Jan 30;11:69. doi: 10.1186/1471-2105-11-69 (PMC2828439; doi:10.1186/1471-2105-11-69)

0.01

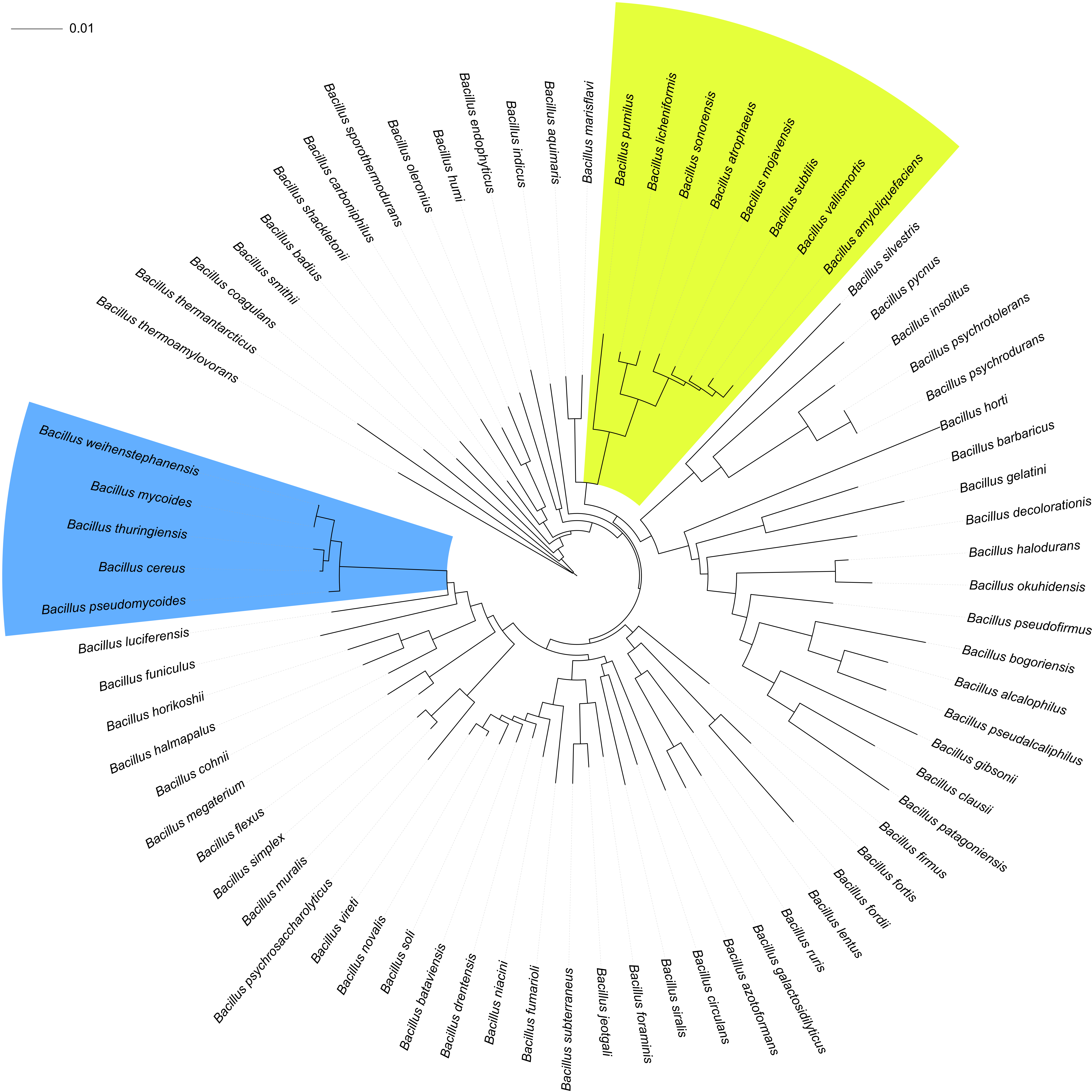

Supplement: Additional file 1 — 16S rRNA gene neighbor-joining tree. Bacillus 16S rRNA gene neighbor-joining tree as constructed by PHYLIP 3.68 and based on sequences selected from the SILVA database. Only the species present in the original data set are visualized. The tree is visualized using the iTol webtool [40]. The Bacillus cereus and Bacillus subtilis groups are coloured in blue and green, respectively. [file 1471-2105-11-69-S1.PDF]

−0.001

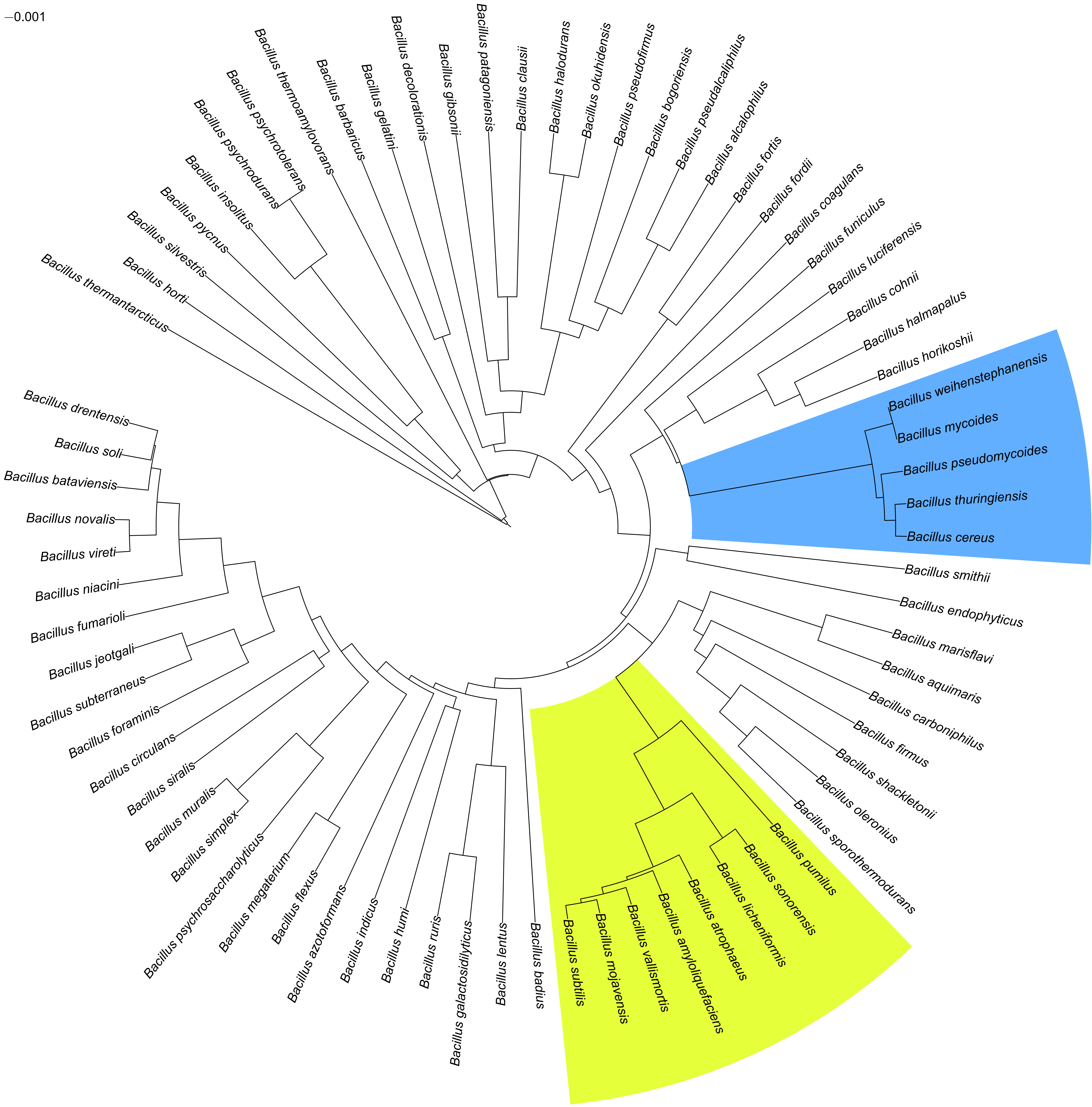

Supplement: Additional file 2 — 16S rRNA gene UPGMA tree. Bacillus 16S rRNA gene UPGMA tree as constructed by PHYLIP 3.68 and based on sequences selected from the SILVA database. Only the species present in the original data set are visualized. The tree is visualized using the iTol webtool [40]. The Bacillus cereus and Bacillus subtilis groups are coloured in blue and green, respectively. [file 1471-2105-11-69-S2.PDF]

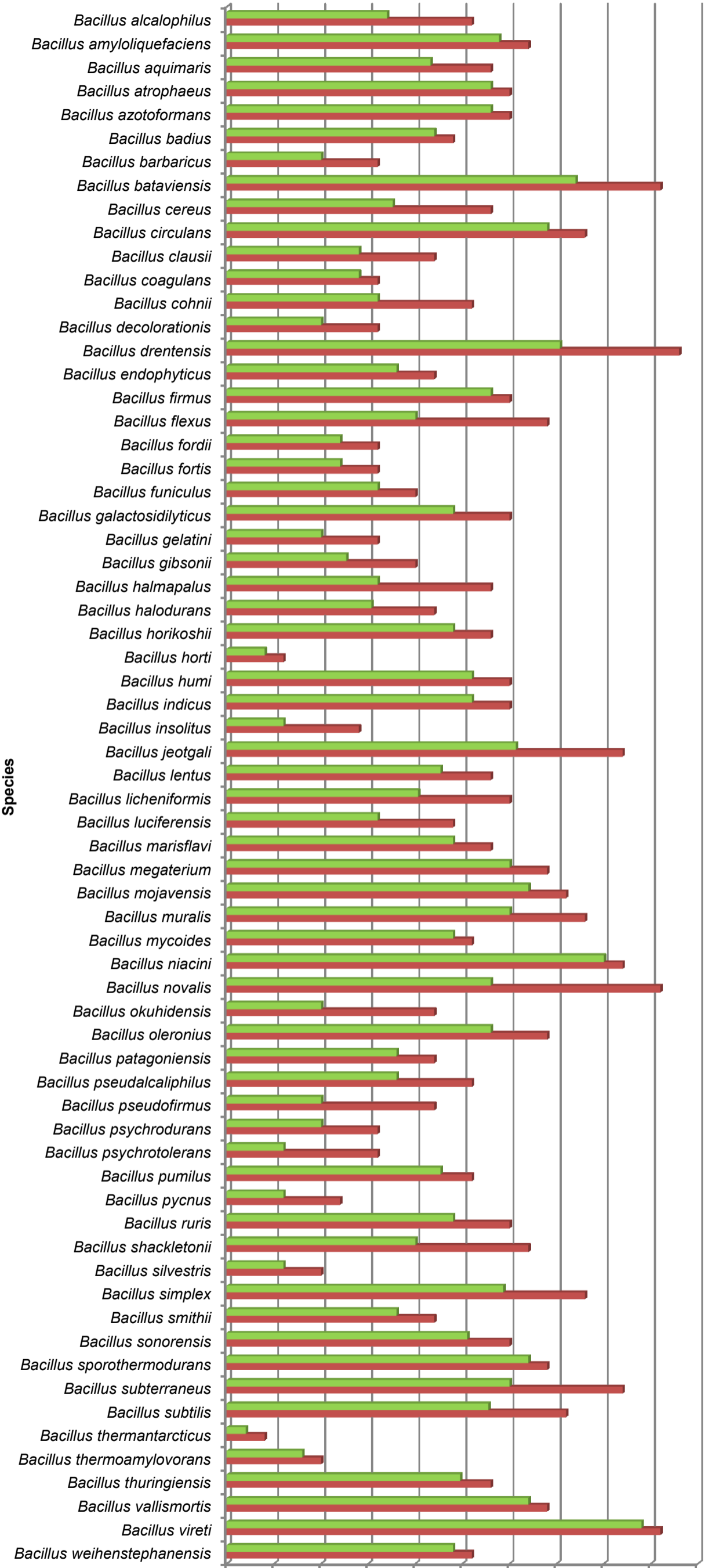

Supplement: Additional file 3 — Average misclassification depth of phylogenetic learning based on a UPGMA tree. The average depth of the misclassified test profiles of each species is visualized for phylogenetic learning based on a UPGMA tree. Depth equals the number of nodes along the classification path until misclassification occurs (the corresponding node included) and corresponds to the green bars. The maximum or correct depth is shown by the red bars. Maximum depth equals the number of nodes along the true phylogenetic path (leaf included). [file 1471-2105-11-69-S3.PDF]
