# Supplementary material for: From learning taxonomies to phylogenetic learning: Integration of 16S rRNA gene data into FAME-based bacterial classification
Source: BMC Bioinformatics. 2010 Jan 30;11:69. doi: 10.1186/1471-2105-11-69 (PMC2828439; doi:10.1186/1471-2105-11-69)
Supplement: Additional file 4 — Table S1 -- Strain list and corresponding 16S rRNA gene accession numbers. List of the 74 considered Bacillus species together with their type strain number and the accession number of the selected 16S rRNA gene sequence. [file 1471-2105-11-69-S4.PDF]

Table S1 - Strain list with according 16S rRNA gene accession numbers
List of the 74 included *Bacillus* species with their type strain number
and the accession number of the selected 16S rRNA gene sequence.

**Species name**	**Strain Number**	**Accession Number**
Bacillus alcalophilus	DSM 485^{T}	X76436
Bacillus amyloliquefaciens	NBRC 15535^{T}	AB255669
Bacillus aquimaris	TF-12	AF483625
Bacillus atrophaeus	JCM9070^{T}	AB021181
Bacillus azotoformans	NBRC 15712^{T}	AB363732
Bacillus badius	NBRC 15713^{T}	AB271748
Bacillus barbaricus	DSM 14730^{T}	AJ422145
Bacillus bataviensis	LMG 21832^{T}	AJ542507
Bacillus bogoriensis	LMG 22234^{T}	AY376312
Bacillus carboniphilus	JCM 9731^{T}	AB021182
Bacillus cereus	CCM 2010^{T}	DQ207729
Bacillus circulans	ATCC 4513^{T}	AY043084
Bacillus clausii	DSM 8716^{T}	X76440
Bacillus coagulans	ATCC 7050^{T}	DQ297928
Bacillus cohnii	DSM 6307^{T}	X76437
Bacillus decolorationis	LMG 19507^{T}	AJ315075
Bacillus drentensis	LMG 21831^{T}	AJ542506
Bacillus endophyticus	CIP 106778^{T}	AF295302
Bacillus firmus	IAM 12464^{T}	D16268
Bacillus flexus	IFO15715^{T}	AB021185
Bacillus foraminis	LMG 23174^{T}	AJ717382
Bacillus fordii	LMG 22080^{T}	AY443039
Bacillus fortis	LMG 22079^{T}	AY443038
Bacillus fumarioli	LMG 17489^{T}	AJ250056
Bacillus funiculus	CIP 107128^{T}	AB049195
Bacillus galactosidilyticus	LMG 17892^{T}	AJ535638
Bacillus gelatini	LMG 21880	AJ551329
Bacillus gibsonii	DSM 8722^{T}	X76446
Bacillus halmapalus	DSM 8723^{T}	X76447
Bacillus halodurans	DSM 497^{T}	AJ302709
Bacillus horikoshii	DSM 8719^{T}	AB043865
Bacillus horti	JCM 9943^{T}	D87035
Bacillus humi	LMG 22167^{T}	AJ627210
Bacillus indicus	DSM 15820^{T}	AJ583158
Bacillus insolitus	DSM 5^{T}	AM980508
Bacillus jeotgali	JCM 10885^{T}	AF221061

Bacillus lentus	NCIMB 8773^{T}	AB021189
Bacillus licheniformis	DSM 13^{T}	X68416
Bacillus luciferensis	LMG 18422^{T}	AJ419629
Bacillus marisflavi	JCM 11544^{T}	AF483624
Bacillus megaterium	IAM 13418^{T}	D16273
Bacillus mojavensis	IFO 15718^{T}	AB021191
Bacillus muralis	LMG 20238^{T}	AJ628748
Bacillus mycoides	ATCC 6462^{T}	AB021192
Bacillus niacini	IFO 15566^{T}	AB021194
Bacillus novalis	LMG 21837^{T}	AJ542512
Bacillus okuhidensis	JCM 10945^{T}	AB047684
Bacillus oleronius	ATCC 700005^{T}	AY988598
Bacillus patagoniensis	DSM 16117^{T}	AY258614
Bacillus pseudalcaliphilus	DSM 8725^{T}	X76449
Bacillus pseudofirmus	DSM 8715^{T}	X76439
Bacillus pseudomycoides	DSM 12442^{T}	AM747226
Bacillus psychrodurans	DSM 11713^{T}	AJ277984
Bacillus psychrosaccharolyticus	ATCC 23296^{T}	AB021195
Bacillus psychrotolerans	DSM 11706^{T}	AJ277983
Bacillus pumilus	DSM 27^{T}	AY456263
Bacillus pycnus	NBRC 101231^{T}	AB271739
Bacillus ruris	LMG 22866^{T}	AJ535639
Bacillus shackletonii	LMG 18435^{T}	AJ250318
Bacillus silvestris	DSM 12223^{T}	AJ006086
Bacillus simplex	DSM 1321^{T}	AJ439078
Bacillus siralis	CIP 106295	AF071856
Bacillus smithii	DSM 4216^{T}	Z26935
Bacillus soli	LMG 21838^{T}	AJ542513
Bacillus sonorensis	BCRC 17416^{T}	EF433411
Bacillus sporothermodurans	DSMZ 10599^{T}	U49078
Bacillus subterraneus	DSM 13966^{T}	AY672638
Bacillus subtilis subsp. *subtilis*	DSM 10^{T}	AJ276351
Bacillus thermantarcticus	DSM 9572^{T}	AJ493665
Bacillus thermoamylovorans	LMG 18084^{T}	L27478
Bacillus thuringiensis	ATCC 10792^{T}	AF290545
Bacillus vallismortis	DSM 11031^{T}	AB021198
Bacillus vireti	LMG 21834^{T}	AJ542509
Bacillus weihenstephanensis	DSM 11821^{T}	AB021199
